# Ethnic minority women prefer strong recommendations to be screened for cancer

**DOI:** 10.1186/s12889-017-4093-2

**Published:** 2017-02-03

**Authors:** Laura A. V. Marlow, Susanne F. Meisel, Jane Wardle

**Affiliations:** Cancer Research UK Health Behaviour Research Centre, Department of Epidemiology & Public Health, UCL, Gower Street, London, WC1E 6BT UK

**Keywords:** Screening, Race, Ethnicity, Inequalities, Recommendation, Literacy

## Abstract

**Background:**

Cancer screening invitations can explicitly recommend attendance or encourage individuals to consider the risks and benefits before deciding for themselves. Public preferences for these approaches might vary. We explored ethnic minority women’s preferences for a strong recommendation to be screened.

**Methods:**

Women aged 30–60 years from Indian, Pakistani, Bangladeshi, Caribbean, African and white British backgrounds (*n* = 120 per group) completed face-to-face interviews with a multi-lingual interviewer. The interview included a question on which approach to screening invitations they would prefer: i) A strong recommendation from the National Health Service (NHS) to go for screening, ii) A statement that the NHS thinks you should go for screening, but it’s up to you to decide, iii) No recommendation. Analyses examined predictors of preference for a strong recommendation.

**Results:**

Preferences varied by ethnicity (*χ* ^2^(5) = 98.20, *p* <.001). All ethnic minority groups had a preference for a strong recommendation to be screened (53–86% across ethnic groups vs 31% white British). Socio-demographic factors (marital status, education and employment), and indicators of acculturation (main language and migration status), contributed to explaining recommendation preferences (*χ* ^2^(5) = 35.95 and *χ* ^2^(3) = 11.59, respectively, both *p* <.001), but did not mediate the ethnicity effect entirely. Self-rated comprehension of written health information did not contribute to the model.

**Conclusions:**

A strong recommendation to participate in cancer screening appears to be important for ethnic minority women, particularly non-English speakers. Future research could explore how to best arrive at a consensus that respects patient autonomy while also accommodating those that would prefer to be guided by a trusted source.

## Background

Increasingly, people are encouraged to take an active role in their healthcare management [1–3] including making informed decisions about preventive health such as cancer screening [4] where the importance of personal choice about whether to participate is now highlighted in policy [5]. However, information required to fully understand the risks and benefits of screening can be complex and it has been argued that the investment of time and effort in reaching a fully informed screening decision could be burdensome for some people [6]. Complicating this further there are numerous aspects of informed decision making, for example the importance of screening decisions being consistent with preferences and values, the need for deliberation about pros and cons of participating in screening and aims to reduce decisional conflict [7].

One facet of informed decision making is ‘role preference’, the extent to which potential service users prefer to take an active role in their healthcare management versus deferring the decision-making to the recommendation of a healthcare provider (sometimes referred to as intellectual outsourcing) [6, 8–10]. There may be a range of opinions regarding the acceptability of a recommendation for screening: It may be perceived as mitigating burden (e.g. by minimising deliberation about information and mitigating decisional conflict) but there may also be concerns regarding apparent coerciveness [6]. This is important in contexts like the UK, which have a “one-size-fits-all” model of communication versus contexts like the US which are more amenable to “shared decision-making” during a face-to-face interaction with a physician [11]. In health systems with centrally organized cancer screening programs (such as in the UK), invitations to screening may be sent without direct involvement of the primary healthcare provider. In this context, empirical evidence for public preferences for a recommendation to attend screening is limited. One qualitative study indicated that women preferred a recommendation to attend mammography screening alongside information on benefits and risks [12]. This matches findings from a recent population-based study of older adults’ attitudes to colorectal cancer screening which showed that most were in favor of a recommendation, alongside information on benefits and harms [13].

There are likely to be a range of preferences for involvement in decision making, but some groups, particularly those with limited capability to process the complex information involved; for example because of having lower literacy and numeracy skills [14, 15], may prefer recommendations. Lack of health literacy (the ability to read and act upon written health information, communicate needs with health professionals, and understand health-related information [16] has commonly been cited as a barrier to informed decision-making [17]. A recent review looking at associations between health literacy and concepts related to informed decision making in the context of colorectal cancer suggested that those with lower health literacy had lower levels of screening knowledge and less positive attitudes towards screening [18]. In a study of Australian adults with low literacy, a bowel cancer screening decision aid increased participants’ knowledge but resulted in reduced uptake of cancer screening, suggesting that after gaining more information people believed the risks did not outweigh the benefits and showing that improvements in knowledge are not always accompanied by increases in screening participation [19].

Ethnic minority status has been associated with poorer health literacy [20, 21]. which could make it difficult for people from ethnic minority backgrounds to read and interpret screening invitations and the written information that accompanies them. This could mean that screening information is not being made accessible to all [22, 23]. In such a situation the individual faced with a screening decision may prefer a strong provider recommendation about cancer screening. Many studies have looked at preferences for decision-making in the context of medical care, with a number of US studies suggesting that some ethnic minority groups prefer to leave decisions about medical care up to their doctor [24, 25]. Not speaking English has been associated with less desire to participate in medical care decisions [26]. Although providing information on the harms and benefits of screening is essential, this could be accompanied with a recommendation. To our knowledge no studies have explored preferences for a recommendation vs IDM in the context of organized cancer screening among ethnic minorities.

In the UK there are three organized cancer screening programme. Each of these invites members of the eligible population (based on age and gender) at regular intervals (usually every 3–5 years) to participate in screening. For bowel screening men and women are sent an FOBT kit through the post to complete and return. For breast screening women are sent an invitation to attend for a pre-set mammography appointment at a local clinic and for cervical screening women are sent an invitation to contact their health care provider and make an appointment for a sample to be taken (usually by a nurse practitioner). On occasion a GP may raise the topic of cancer screening during a consultation about another health issue if they can see on the patients records that they are overdue. The current study was embedded in a wider survey of women aged 30–60 years. All of these women were eligible for cervical screening and some of them (those aged 50–60 years) were also eligible for breast screening. The aim of this paper was to explore whether preferences for a screening recommendation varied by ethnicity and if socio-demographic factors, indicators of acculturation, English proficiency or health literacy might mediate this association.

## Methods

Data were collected by Ethnic Focus, a market research company that uses quota sampling to recruit participants from ethnic minority groups from across England. We commissioned Ethnic Focus to recruit 720 women aged 30–60 years from Indian, Pakistani, Bangladeshi, African, Caribbean and White British backgrounds (120 women from each ethnic group).

### Sampling

Ethnic Focus maintains a list of sampling points based on census information about the concentrations of different ethnic minority groups within each post-code sector. The list is updated biannually, and at the time of recruitment contained 370 sampling points. Sampling points (*n* = 35) were randomly selected and inspected to ensure they represented areas of high, medium and low concentrations of ethnic minority residents. Multi-lingual interviewers visited properties within each sampling point looking for eligible participants (determined by age, gender and ethnicity). If an eligible participant lived in a household, an interview was carried out or the interviewer returned later. Three attempts were made to contact an eligible participant before they were counted as a non-responder. No incentive was offered for participation. The study was considered exempt from needing approval from the UCL Research Ethics Committee because data were collected anonymously.

### Materials

Women completed a series of standard questions with a female multi-lingual interviewer. The interviewer was employed by Ethnic Focus and was not part of the research team but was fully briefed about the aims of the study. Questions were translated into the most common languages prior to data collection and checked for consistent meaning by bi-lingual researchers. Interviews were carried out in the woman’s main language. This was part of a wider study with a focus on attitudes to cancer and cervical cancer screening (full questionnaire available on request).

#### Recommendation preferences

Preferences for screening recommendations were assessed using a single item with three response options: “Imagine you were being invited to go for cancer screening as part of the NHS screening programme. In the information you receive from the NHS, would you prefer: i) A strong recommendation from the NHS to go for screening; ii) A statement that the NHS thinks you should go for screening but that it’s up to you to decide; iii) No recommendation – it’s up to you to decide whether or not to go for screening”. This item was based on previous work assessing preferences for a recommendation in the context of colorectal cancer screening [13]. No information was provided about cancer screening until after the question about preferences for a recommendation. Therefore these findings are based on women’s preconceived views about the voluntary nature of screening in the UK.

#### Socio-demographic factors

Women were asked ‘What is your age?’ (open response box), What is your marital status? (Single, Married, Cohabiting, Divorced or separated, Widowed), What is the highest educational qualification you have obtained? (No formal qualifications, O-levels, ONC or BTec, A-levels or highers, higher education below degree, degree or higher, still studying, other) and ‘Are you currently…’ (Working as an employee, self-employed or freelance, working in the family business, away from work ill or on maternity leave, doing any other work, retired, student, looking after the home or family, long-term sick or disabled, other). These questions were all taken from the 2011 UK Census [27], a mandatory survey of all UK residents carried out every 10 years. Ethnicity was also assessed using a question from the census, with women asked ‘What is your ethnic group?’ and offered 18 response options under five major headings (White, Asian or Asian British, Mixed or multiple ethnic groups, Black/African/Caribbean/Black British, Other ethnic group).

#### Indicators of acculturation

We used migration status, main language spoken, and length of residency in the UK as indicators of acculturation. Similar variables were used in a large cohort study of women [28]. Questions were taken from the 2011 census questions [27]. To establish l*ength of residency* in the UK, we subtracted their year of arrival from the year of data collection. *Migration status* was determined using women’s year of arrival in the UK and current age, recoding this as: born in the UK; migrated <18 years old; migrated ≥18 years old. Women also reported their *main language* as ‘English’ or ‘other’.

#### English proficiency

Women who reported a main language other than English were asked “how well can you: i) read English, and ii) speak English (response options: very well/well/not well/not at all). These probe ability to read and speak English and were taken from the UK-Censes. In the census they are referred to as English proficiency.

#### Health literacy

The survey format meant assessment of health literacy using a validated tool was not possible (these tools take a significant amount of time to complete and have not been translated and validated into the languages we needed). Instead, two single items were adapted from the European Health Literacy Project [29] assessing self-rated ability to communicate with the GP: “How easy do you find it to understand what the GP says to you?” and self-rated ability to comprehend written health materials: “How easy do you find it to understand leaflets and letters about your health?” Response options for both questions were: very easy/fairly easy/fairly difficult/very difficult). Questions were asked to all women regardless of their main language. These questions were chosen because we felt they best tapped aspects relevant to how screening invitations are sent (as written materials) or how concerns might be discussed (with a GP).

### Analyses

Recommendation preferences were examined individually and recoded into a binary variable indicating a preference for a “strong recommendation” (response option i) versus “it’s up to you” (response option ii or iii). Logistic regression analyses were first used to explore the influence of ethnicity, migration status and main language spoken on recommendation preferences. We then considered the role of other socio-demographic variables and self-rated rated ability to communicate with the GP and comprehend written health materials, before and after adjusting for ethnicity and language in multivariate models.

## Results

Overall, 1116 eligible interviewees were approached in order to complete 720 interviews (response rate = 65%). The response rate was higher for white British and Indian women (71%) than Pakistani, Bangladeshi, African and Caribbean women (61–63%). Demographic characteristics varied by ethnic group and were broadly in line with national data. The majority of Indian, Pakistani and Bangladeshi women were married (82, 93 and 96%) compared with 62/64/36% of African, white British and Caribbean women. Caribbean, White British, Indian and African women were more likely to be working than Pakistani and Bangladeshi women (54–66% compared with 40 and 28%). All the Indian and white British women had at least some educational qualifications, whereas around 18–24% of African, Caribbean, Bangladeshi and Pakistani women had no formal qualifications.

**Fig. 1:**
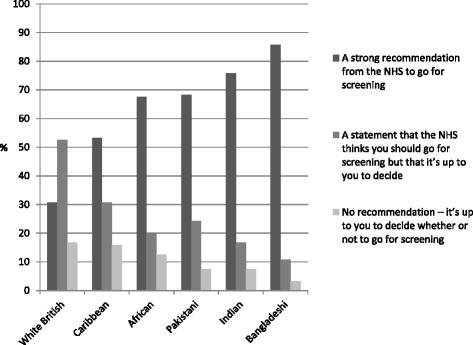
Recommendation preferneces for each ethnic group

Overall, 64% of women wanted a strong recommendation to be screened, 26% wanted a statement saying the NHS thinks you should go but it’s up to you to decide, and 11% wanted no recommendation - preferring that it was entirely up to them to decide. Recommendation preferences varied by ethnicity (*χ* ^2^(5) = 98.20, *p* <.001) with women from each of the ethnic minority groups more likely to want a strong recommendation to be screened than the white British women (see Fig. 1). The difference was smallest for Caribbean women (OR = 2.56, 95% CI: 1.51-4.35 compared with white British women), and most pronounced for Bangladeshi women (OR = 13.59, 95% CI: 7.15–25.85 compared with white British women). See Table 1.

**Table 1:** Correlates of preferring a strong recommendation to be screened (univariate)

	Strong recommendation %	Odds ratio [95% confidence interval]
Ethnicity		
White British (*n* = 120)	30.8	1.00
Caribbean (*n* = 120)	53.3	2.56 [1.51–4.35]***
African (*n* = 120)	67.5	4.66 [2.71–8.03]***
Pakistani (*n* = 120)	68.3	4.84 [2.80–8.36]***
Indian (*n* = 120)	75.8	7.04 [3.98–12.45]***
Bangladeshi (*n* = 120)	85.8	13.59 [7.15–25.85]***
Age		
30–40 (*n* = 296)	62.5	1.00
40–50 (*n* = 262)	66.0	1.17 [0.82–1.65]
50–60 (*n* = 162)	61.7	0.97 [0.65–1.44]
Marital		
Not married (*n* = 202)	49.5	1.00
Married (*n* = 518)	69.1	2.28 [1.64–3.18]***
Employment		
Working (*n* = 367)	53.4	1.00
Not working (*n* = 353)	74.2	2.51 [1.83–3.44]***
Education		
Above GCSE-level (*n* = 375)	50.7	1.00
GCSEs (*n* = 85)	63.5	1.70 [1.59–4.16]***
No formal qualifications (*n* = 102)	72.5	2.57 [1.59–4.16]*
Other (*n* = 158)	88.6	7.57 [4.45–12.88]***
Migration status		
Born in the UK (*n* = 314)	44.9	1.00
Under 18 years (*n* = 111)	67.6	2.56[1.62–4.03]***
Over 18 years (*n* = 295)	82.0	5.60 [3.86–8.12]***
Main Language		
English (*n* = 431)	49.2	1.00
Other (*n* = 289)	85.1	5.91 [4.06–8.60]***
Ability to speak English		
Main language English (*n* = 431)	49.2	1.00
Speak English well/very well (*n* = 89)	83.1	5.10 [2.84–9.16]***
Do not speak English well/at all (*n* = 200)	86.0	6.35 [4.08–9.87]***
Ability to read English		
Main language English (*n* = 431)	49.2	1.00
Read English well/very well (*n* = 62)	85.5	6.08 [2.93–12.64]***
Do not read English well/at all (*n* = 227)	85.0	5.86 [3.89–8.84]***
Comprehension of health information		
Very Easy (*n* = 355)	52.4	1.00
Fairly easy (*n* = 101)	55.4	1.13 [0.73–1.76]
Fairly difficult (*n* = 208)	79.3	3.49 [2.35–5.18]***
Very difficult (*n* = 56)	91.1	9.27 [3.61–23.77]***
Ability to understand the GP		
Very Easy (*n* = 380)	55.0	1.00
Fairly easy (*n* = 183)	69.9	1.90 [1.31–2.77]***
Fairly difficult (*n* = 84)	75.0	2.45 [1.44–4.19]***
Very difficult (*n* = 73)	79.5	3.16 [1.73–5.78]***

*p <.05, ***p <.001

### Socio-demographic factors

Age was not associated with preference for a strong recommendation to be screened, but married women preferred a strong recommendation (69 vs 50% of unmarried women, *p* <.001) as did women who were not working (79 vs 53% of women who were working, *p* <.001). Among women with educational qualifications beyond the basic level, 51% preferred a strong recommendation, while those who had limited or no formal qualifications were more likely to want a strong recommendation to be screened (around 70%, *p* <.05). Women with an ‘other’ qualification (all of whom were born outside the UK) were also much more likely to want a strong recommendation (89%).

A multivariate model, which included marital status, employment, education, and ethnicity significantly predicted preference for a strong recommendation to be screened (*χ* ^2^(10) = 134.12, *p* <.001) explaining 23% of the variance (Nagelkerke R^2^ = .233). Adding socio-demographics explained a further 6% of the variance (Step 2: *χ* ^2^(5) = 35.95, *p* <.001), however all ethnic minority groups remained significantly more likely to want a strong recommendation for screening (see Table 2). The odds ratios for each South Asian group reduced by around half, but for the African and Caribbean women there was virtually no difference from the basic model.

**Table 2:** Multivariate logistic regression analyses to determine which variables confound ethnicity

	Step 1: Unadjusted ethnicity only	Step 2: Adjusted for socio-demographics^b^	Step 3: Adjusted for socio-demographics; acculturation; language	Step 4: Adjusted for socio-demographics, acculturation, language; comprehension of health information
Ethnicity^a^				
White British	1.00	1.00	1.00	1.00
Caribbean	2.56 [1.51–4.35]***	2.60 [1.47–4.60]*	2.24 [1.18–4.24]*	1.80 [0.89–3.63]
African	4.66 [2.71–8.03]***	4.31 [2.46–7.54]**	2.90 [1.50–5.62]**	2.64 [1.34–5.23]**
Pakistani	4.84 [2.80–8.36]***	2.90 [1.59–5.31]**	2.06 [1.09–3.91]*	1.83 [0.92–3.64]
Indian	7.04 [3.98–12.45]***	4.43 [2.36–8.32]***	3.17 [1.64–6.14]**	2.69 [1.34–5.43]**
Bangladeshi	13.59 [7.15–25.85]***	6.76 [3.33–13.71]***	5.22 [2.51–10.84]***	4.38 [2.01–9.51]***
Step: *χ* ^2^(df), *p*-value	-	35.95 (5), <.001	11.59 (3), .009	4.20 (3), .240
Full model: *χ* ^2^(df), *p*-value	98.20 (5), <.001	134.12 (10), <.001	145.72 (13), <.001	149.92 (16), <.001
Nagelkerke R^2^	.175	.233	.251	.257

*p <.05, **p <.01, ***p <.001

^a^Odds Ratio [95% Confidence Interval]

^b^marital status, employment and education

### Indicators of acculturation

Next we explored indicators of acculturation as variables that could potentially explain the influence of ethnicity on preference for a strong recommendation. In univariate analyses, women who were born outside the UK were more likely to want a strong recommendation than those born in the UK (78 vs 45%, *p* <.001), and this association was more pronounced for women who had migrated as an adult (OR = 5.60, 95% CI: 3.86–8.12), than as a child (OR = 2.56, 95% CI: 1.62–4.03). Length of residency in the UK entered as a continuous variable (mean 24.51, range 1–60 years) was not significantly associated with recommendation preferences (OR = 0.99, 95% CI: 0.96–1.01). Women who did not speak English as their main language were more likely to want a strong recommendation to be screened (85 vs 49%, *p* <.001). Including migration status and main language made a small but significant contribution to the model (Step 3: *χ* ^2^(3) = 11.59, *p* = .009), explaining an additional 1.8% of the variance (see Table 2), and reduced the odds ratios for each ethnic group (mean = 1.08 reduction in OR, range:0.36–1.54), suggesting partial mediation.

### English proficiency and health literacy

Among women whose main language was not English, self-reported ability to speak or read English did not seem to influence recommendation preferences. Health literacy was strongly correlated with ability to read and speak English (*r* = .87 and *r* = .86 respectively, *p* <.001). Most women who spoke English found it easy to understand leaflets and letters about their health, while all of those who did not speak English well/at all found this difficult. Among women whose main language was not English, but reported speaking English well (*n* = 89), 43% found it difficult to understand leaflets and letters about their health. There appeared to be a gradient in health literacy, with women who found it more difficult to understand written health information more likely to want a strong recommendation to be screened with a similar pattern for the variable assessing how easy it is to understand health information. Including health literacy did not significantly add to the model (step 4: *χ* ^2^(3) = 4.20, *p* = .240, Nagelkerke R^2^ = .257). However, the odds ratios were further reduced (mean = 0.45 reduction in OR, range:0.23–0.84) and were no longer significant for Pakistani and Caribbean women; suggesting some mediation.

## Discussion

This study is the first to show that women from an ethnic minority background prefer to receive a strong provider recommendation to attend cancer screening. Perhaps surprisingly, this effect persisted for most ethnic minorities even after controlling for English proficiency, and self-reported comprehension of health information materials. This suggests that the preference for a recommendation to be screened is not entirely driven by understanding of health information materials.

Women’s preferences may spur from unfamiliarity with the informed choice approach which is advocated in Western societies [30]. This may also explain why we found that women who migrated as adults were more likely to prefer a recommendation than those who migrated to the UK as a child. Alternatively, it is possible that women in more traditional societies see doctors as experts whose judgment is to be trusted, and thus prefer a recommendation. A recent study exploring Breast Health Practices among Asian women in Canada suggested doctors are held in very high regard and women felt BSE or mammograms were not necessary if they had not been recommended by a doctor [31]. Future research could explore these findings in more detail. It is possible that tailoring information for ethnic minority groups to make clear that health professionals recommend screening may help to satisfy preferences for a recommendation, as long as risks and benefits are also communicated. Recommendations in the context of organized screening programmes could be made by including statements of endorsement in information materials, for example a banner at the top of the invitation letter stating ‘Your GP Practice, supports Screening’ improved uptake in a recent trial of bowel screening interventions [32]. However, for those who do not read, GP endorsement messages may be best delivered using audio/video media. Practitioners who are working in geographic areas with a high proportion of ethnic-minority men and women in the population may wish to incorporate recommendations into additional screening reminders.

Much valuable work has already been done to overcome language barriers in clinical care by providing health information leaflets in languages other than English, and by using medical interpreters; with both showing some positive impact [33–35]. However, the current results suggest that this is only a first step. It will be vital to improve communication about cancer screening to foster individual engagement with health information. One way could be to develop cancer communication materials and decision aids that take into account the broader cultural context in collaboration with stakeholders from ethnic minorities.

This study had a number of strengths. The survey was conducted by an external survey company, which reduces the risk of researcher bias. The large sample size meant that the study was sufficiently powered; increasing confidence in the current findings. Questions were translated into the respondents’ first language using qualified translators to enhance understanding and overcome language barriers. The survey was on the wider topic of cancer screening, and participants consented to the entire survey which minimized the risk of response bias on this specific topic (although they could withdraw at any time).

The study also had important limitations. Although the item assessing preference for a screening recommendation had been used in previous research, it was based on a hypothetical scenario, which may have been difficult to understand for some respondents. Furthermore, because of constraints on the survey format, no detail was given about the process of screening, or the information that would be provided; it is therefore possible that some women thought they would not receive any information about screening at all if they opted for not receiving a strong recommendation. We also did not clarify in the question what we mean by a ‘strong’ recommendation (as opposed to a weak recommendation or just a recommendation) and this would therefore have been left open to interpretation by each respondent. It is possible that lack of knowledge about screening could confound preferences for screening recommendations, but since we did not measure this we cannot explore the role it plays. All questions were ‘closed’, so it was not possible to investigate the reasons behind participants’ responses in further detail. This could be done in future research. The survey format meant a full assessment of health literacy using a validated tool like the Tofla was not possible. Instead we used two items that we felt were relevant to how women might understand information communicated to them about screening; self-rated comprehension of written health materials and ability to understand the GP. Although quota sampling was employed to achieve sufficient participant numbers from each ethnic background, this method lacks the rigor of random sampling and recruitment from areas with high concentrations of ethnic minorities may exaggerate ethnic differences; therefore, results may not generalize to the wider ethnic minority population. But random sampling of multiple ethnic minority groups would be very expensive.

## Conclusions

These results give a first indication that some ethnic groups may prefer recommendations in cancer screening. Providing a recommendation for ethnic minority women is likely to be important, but ethnic minority women also need to be empowered and encouraged to engage with information. Future research could explore how to best arrive at a consensus that respects patient autonomy while also accommodating those that would prefer to be guided by a trusted source.
